# Optimization of spring parameters by using the Bees algorithm for the foldable wing mechanism

**DOI:** 10.1038/s41598-022-26361-1

**Published:** 2022-12-19

**Authors:** Murat Sahin, Zafer Kulunk

**Affiliations:** 1 Control Systems Department, Roketsan Inc., 06780 Elmadag, Ankara, Turkey; 2 System Engineering Department, Roketsan Inc., 06780 Elmadag, Ankara, Turkey

**Keywords:** Aerospace engineering, Mechanical engineering, Statistics

## Abstract

In this study, the design of the torsion and compression springs of the foldable wing mechanism used in the missile is considered an optimization problem. After the missile leaves the tube, the wings waiting in a closed state must be opened and fixed within a specific time. The study it is aimed to maximize the energy stored by the springs so that the wing can be opened in a minimum time. In this context, the energy equation in both publications is defined as the objective function in the optimization process. Wire diameter, coiling diameter, coiling number, and deflection parameters required for spring design were determined as optimization variables. There are geometrical constraints for the variables due to the dimensions of the mechanism and there are also safety factor constraints due to the loads to which the springs are exposed. The Bees Algorithm (BA) was used to solve this optimization problem and perform the spring design. The energy values obtained with BA were better than the values obtained with the Design of Experiment (DOE) study before. The springs and mechanism designed with the parameters obtained from the optimization were first analyzed in the ADAMS program. Afterward, experimental tests carried out by integrating the produced springs into the actual mechanism. As a result of the tests, it was observed that the wing opened at approximately 90 ms. This value is well below the project target of 200 ms. In addition, there is only a 16 ms difference between the analysis and the experimental results.

## Introduction

In aircraft and marine vehicles, folding mechanisms have critical tasks. These systems are used in morphing aircraft and conversion works to improve flight performance and control. Depending on the flight mode, the wings are folded and reopened in different ways to reduce aerodynamic effects^1^. This situation can be compared to the wing movements of some birds and insects during routine flight and diving^2^. Similarly, gliders fold and unfold in underwater vehicles to reduce hydrodynamic effects and maximum driving performance^3^. Another task of the mechanisms is to provide volume advantages to the systems such as folding the propellers of helicopters during storage and transportation^4^. The missiles’ wings are also folded to reduce storage space. This way, more missiles can be placed in a smaller area on launcher system^5^. The components used effectively in folding and unfolding are usually springs. At the moment of folding, energy is stored in them, released at the moment of unfolding. Thanks to their flexible structure, the energy stored and released becomes equal. Springs are mainly designed for the system, and this design is an optimization problem^6^. Because, although it includes different variables such as wire diameter, coiling diameter, coiling number, helix angle, and material type, there are also criteria such as minimizing mass, volume, stress distribution, or having maximum energy^7^.

This study reveals the design and optimization of the springs of the foldable wing mechanism used in a missile system. While inside the launch tube before the flight, the wings remain folded over the missile surface, and after exiting the tube, they open within a specific time and remain locked on the surface. This process is critical for the missile to function correctly. In the folding mechanism designed in the study, the wing's opening is performed by the torsion spring, while the locking operation is performed by the compression spring. In order to design suitable springs, it is necessary to perform an optimization process. There are different applications in the literature within the scope of the optimization of springs.

Paredes et al.^8^ determined maximize the maximum fatigue life factor as the objective function for the helical spring design and used the quasi-Newton approach as the optimization method. The variables in the optimization were determined as wire diameter, coiling diameter, number of turns, and spring length. Another parameter in spring design is the material used in its production. Therefore, it is considered in design and optimization studies. Zebdi et al.^9^ set the target of maximum stiffness and minimum weight in the objective function in a study where the weight factor is essential. They determined the spring material and geometric properties as variables in this context. They used the genetic algorithm as an optimization method. In automotive, the weights of materials are effective in many areas, from vehicle performance to fuel consumption. Minimizing weight in optimizing coil springs used for suspension is a famous study^10^. Bakhshesh and Bakhshesh^11^ determined the materials such as E-glass, Carbon, and Kevlar as a variable in their work in the ANSYS environment and aimed at minimum weight and maximum tension capacity among different composite designs for the suspension springs. The production processes are essential in the design of composite springs. Therefore, in the optimization problem, different variables, such as production methods, steps performed during the process, and the sequence of these steps come into play^12,13^. The system's natural frequency should be considered in springs designed for dynamic systems. It is recommended that the first natural frequency of the spring be at least 5–10 times greater than the natural frequency of the system to avoid resonance^14^. Taktak et al.^7^ chose to minimize the spring-mass and maximize the first natural frequency as the objective function in the helical spring design. They used Patternsearch, Interior point, Active Set, and Genetic algorithm methods in the Matlab Optimization Tool. One of the parts of spring design studies is analysis study, and the finite element method is prevalent in this field^15^. Patil et al.^16^ developed an optimization technique to reduce the weight of the helical compression spring using the analytical process and checked the analytical equations with the finite element method. Another criterion that will increase spring's utility is increasing the energy it can store. This situation also ensures that the spring retains its usefulness over long periods. Rahul and Rameshkumar^17^ aimed to reduce the spring volume and increase the strain energy in the helical coil spring design used in automotive. They also used genetic algorithms in their optimization studies.

It is seen that the parameters in the optimization studies vary according to the systems. In general, stiffness and shear stress parameters are essential in systems where the load they carry is determinant. Material selection is included in weight-constrained systems with these two parameters. On the other hand, the natural frequency is checked to avoid resonance in highly dynamic systems. In systems where utility is significant, energy is maximized. In optimization studies, while analysis studies are carried out with FEM, it is seen that meta-heuristic algorithms such as genetic algorithm^14,18^ and gray wolf algorithm^19^ are used together with classical Newton methods within the scope of determining the parameters. Meta-heuristic algorithms have been developed inspired by the adaptation method of nature, and they approach the optimum in a short time, especially with the power of the population^20,21^. With the random distribution of the population in the search area, they avoid the local optimum and head toward the global optimum^22^. For this reason, it has been frequently used in the context of real industrial problems in recent years^23,24^.

The critical situation for the folding mechanism designed in this study is that the wing, which is in the closed position before the flight, opens before a specific time after exiting the tube. After that, the locking elements lock the wings. Therefore, the flight dynamics will not affect the springs directly. In this context, as the goal of optimization, it was determined to maximize the energy stored to accelerate the spring's movement. Coiling diameter, wire diameter, coiling number, and deflection were determined as optimization parameters. Due to the small size of the springs, weight was not considered a target. Therefore, the material type was defined as fixed. As critical constraint, safety factors were determined in order not to deform mechanically. In addition, dimensional limits for the variables are included in terms of the volumes of the mechanism. BA, a meta-heuristic method, was chosen as the optimization method. BA was preferred because of its flexible and simple structure and its success in mechanical optimization studies^25^. In the second part of the study, detailed mathematical expressions are within the scope of the basic structure of the folding mechanism and the spring design. The third section contains the optimization algorithm and optimization results. In the fourth chapter, there are analyzes made in the ADAMS program. The suitability of the springs was analyzed before production. The last section contains experimental results and images of the tests. The results obtained in the study were also compared with the authors' previous work with the DOE Method.

## Foldable wing mechanism and springs

### Foldable wing mechanism

**Figure 1:**
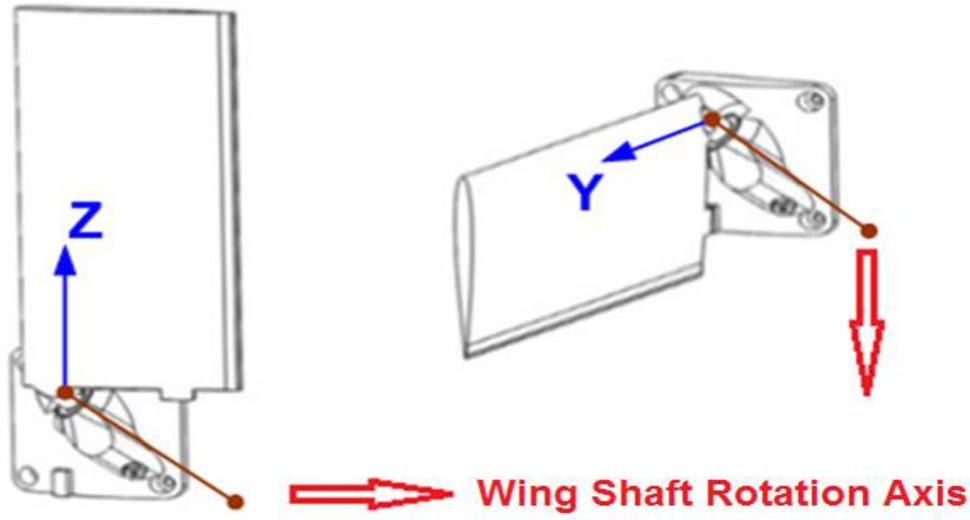
Folded and deployed positions of the missile wing from left to right.

The wing designed in this study should be folded onto the missile’s surface. The wing rotates around an axis from the folded to the deployed position. For this reason, a specific mechanism has been designed. Figure 1 shows folded and deployed configurations in the missile’s coordinate system^5^.

**Figure 2:**
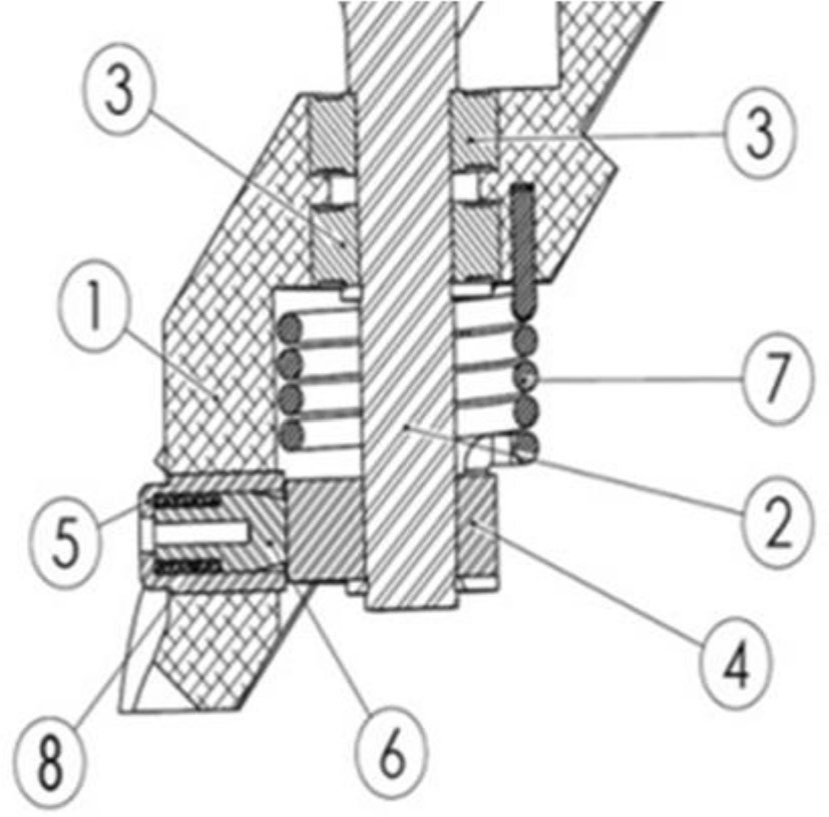
Cross sectional view of the mechanism and components orientation.

Figure 2 shows the section view of the mechanism. The mechanism consists of several mechanical components: (1) main casing, (2) wing shaft, (3) bearings, (4) locking casing, (5) locking bushing, (6) locking pin, (7) torsion spring, and (8) compression spring. The wing shaft (2) is connected to a torsion spring (7) through a locking casing (4). These three parts rotate simultaneously after the missile leaves. With this rotational movement, the wing is deployed to its final position. After that, pin (6) is activated by a compression spring (8) which locks the whole mechanism of the locking casing (4)^5^.

### Mathematical equations of springs

Elasticity modulus (E) and shear modulus (G) are the critical parameters for the spring design. In this study, a high-carbon spring wire (Music wire ASTM A228) has been chosen as a spring material. The other parameters were the wire diameter (d), mean coiling diameter (Dm), coiling number (N), and deflection of springs (xd for compression spring and θ for torsion spring)^26^. The stored energies for compression ${(SE}_{x})$ and torsion (${SE}_{\theta }$) springs can be calculated using Eqs. (1) and (2)^26^. (The Shear Modulus (G) value of the compression spring is 83.7E9 Pa, and the Elasticity Modulus (E) value of the torsion spring is 203.4E9 Pa.)

1 $${SE}_{x}=({ d}^{4} G{ xd}^{2})/(16{ Dm}^{3} N)$$

2 $${SE}_{\theta }=({d}^{4} E {\theta }^{2} \pi /180)/(7776 Dm N)$$

The system’s mechanical dimensions directly determine the springs’ geometric constraints. In addition, the conditions to which the missile will be exposed should be considered. These factors determine the limits of the spring parameters. Another important constraint is the safety factor. The determination of safety factors is detailed in Shigley et al*.*^26^. The safety factor for compression (SFC) springs is defined as the division of maximum allowable stress by stress at solid length. SFC can be calculated using Eqs. (3), (4), (5), and (6)^26^. (For the spring material used in this study, ${S}_{sy}=980 MPa$). F indicates the force in the equations, and K_B_ indicates the Bergstrasser factor^26^.

3 $$SFC=\frac{{S}_{sy}(Max.allowable stress)}{{T}_{s}(Stress at solid length)}$$

4 $${T}_{s}={K}_{B}\frac{8*F*Dm}{\pi *{d}^{3}}$$

5 $${K}_{B}=\frac{\left(4*\frac{Dm}{d}\right)+2}{\left(4*\frac{Dm}{d}\right)-3}$$

6 $$F=xd*\frac{{d}^{4}*G}{8*{Dm}^{3}*N}$$

The safety factor for torsion (SFT) spring is defined as the division of M by k. SFT can be calculated from Eqs. (7), (8), (9), and (10)^26^. (For the material used in this study, ${S}_{y}=1600 \mathrm{MPa}$). In the equations, M is used for the moment, ${k}^{^{\prime}}$ for the spring rate (torque/turn), and K_i_ for the stress correction factor.

7 $$SFT=\frac{M*360}{{k}^{^{\prime}}}$$

8 $$M=\frac{\pi *{d}^{3}*{S}_{y}}{32*{K}_{i}}$$

9 $${K}_{i}=\frac{4*{\left(\frac{Dm}{d}\right)}^{2}-\frac{Dm}{d}-1}{\left(4*\frac{Dm}{d}\right)*(\frac{Dm}{d}-1)}$$

10 $${k}^{^{\prime}}=\frac{{d}^{4}*E}{\mathrm{10,8}*Dm*N}$$

## BA and optimization of the springs parameters

### Objectives and problem formulation

The main purpose of optimization in this study is to maximize the energy of the springs. The objective function is formulated to find $\overrightarrow{\{X\}}$, which maximizes $f(X)$. ${f}_{1}(X)$ and ${f}_{2}(X)$ represent energy functions for compression and torsion spring, respectively. The design variables and functions for optimization are shown in the following equations.

11 $$\overrightarrow{\{{X}_{1}\}}= {\{d,Dm,N,xd\}}^{T}$$

12 $$\overrightarrow{\{{X}_{2}\}}= {\{d,Dm,N,\theta \}}^{T}$$

13 $$max {f}_{1}\left(X\right)= \mathrm{compression }{\mathrm{spring}}^{\mathrm{^{\prime}}}s energy= {SE}_{x}$$

14 $$max {f}_{2}\left(X\right)= \mathrm{torsion }{\mathrm{spring}}^{\mathrm{^{\prime}}}s energy= {SE}_{\theta }$$

The various constraints imposed on the spring design are given in the following equations. Equations (15) and (16) represent the safety factors for compression and torsion springs, respectively. In this study, SFC should be greater than or equal to 1.2, and SFT should be greater than or equal to θ^26^.

15 $${g}_{1}\left(X\right), SFC-1.2 \ge 0$$

16 $${g}_{2}\left(X\right), SFT- \theta \ge 0$$

The lower and upper limits of the spring parameters are given in Table 1.

**Table 1:** Parameters of the compression spring.

Parameters	Compression spring		Torsion spring	
	Minimum	Maximum	Minimum	Maximum
Wire diameter (d)	0.3 mm	0.5 mm	1 mm	1.7 mm
Coiling diameter (Dm)	3.1 mm	3.6 mm	15 mm	21 mm
Coiling number (N)	8	12	3	7
Deflection (xd)	6 mm	9 mm	–	–
Deflection ($\theta$)	–	–	130°	170°

### BA implementation

**Figure 3:**
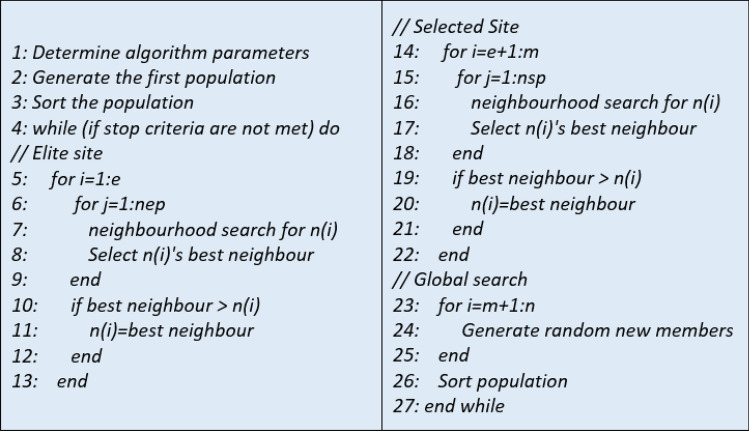
Pseudo-code of BA.

BA was inspired by the pollen-seeking strategies of honey bees^27^. The search method of honey bees is based on sending more collectors to fertile pollen fields and sending fewer collectors to less fertile fields. In this way, the highest efficiency is obtained from the bee population. On the other hand, scout bees continue to look for new pollen areas, and if there are more productive areas than the previous ones, many foragers are directed to this new area^28^. BA consists of two parts: local and global search. In local search, more neighborhoods are searched close to the minimum values (Elite site), as in honey bees, while fewer searches are done in other sites (Best site or Selected Site). Random searches are performed in the global search section, and if good values are found, these sites are transferred to the local search section in the next iteration. The algorithm contains some parameters: number of scout bees (n), number of local search sites (m), number of elite sites (e), number of foraging bees in the elite site (nep), number of foraging bees in the best site (nsp), neighborhood size (ngh) and the number of iterations (I)^29^. The pseudo-code of BA is given in Fig. 3.

**Figure 4:**
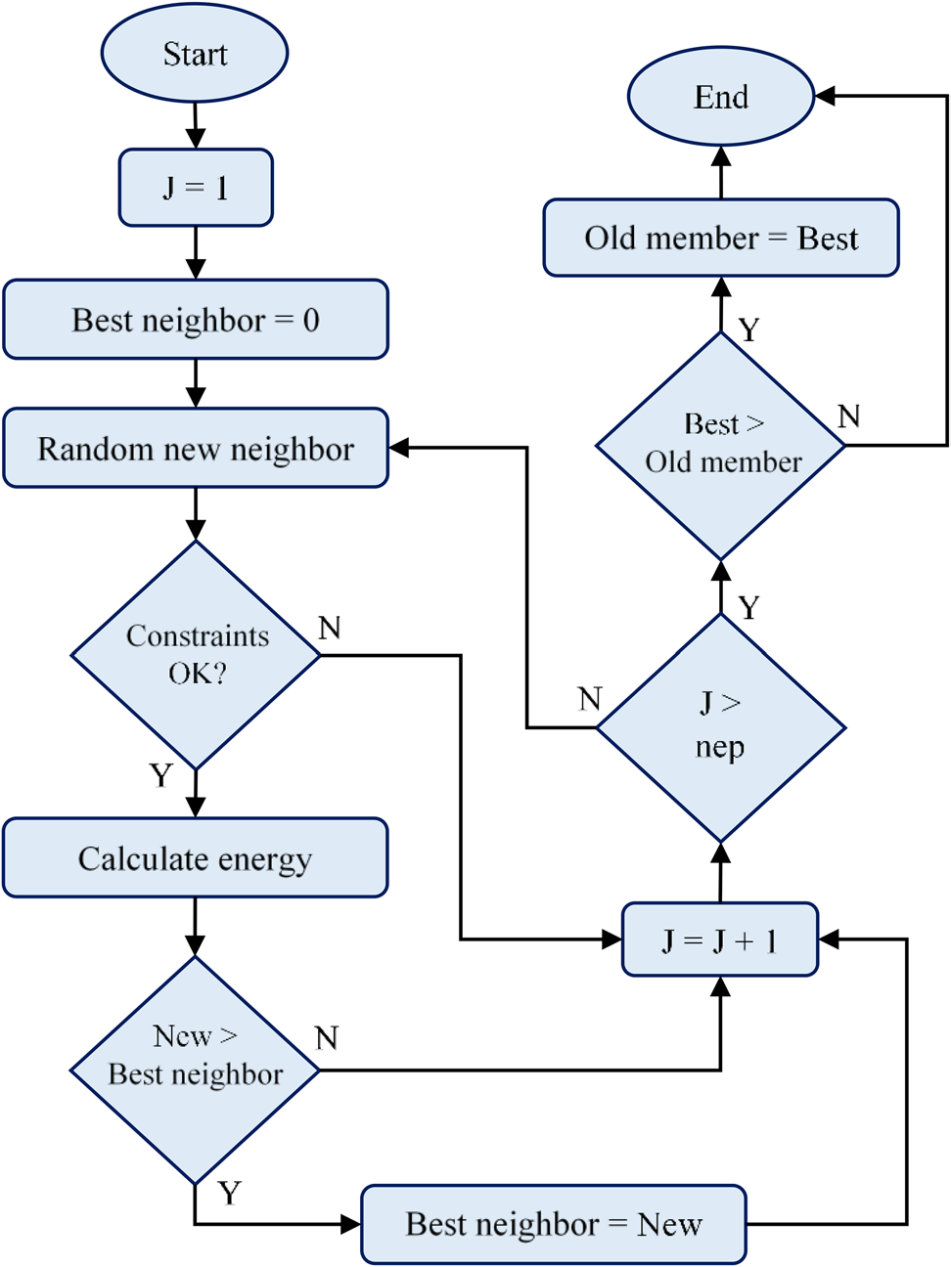
The flowchart of the local search.

The algorithm tries to find the highest values for ${f}_{1}(X)$ and ${f}_{2}\left(X\right)$ within the constraints of ${g}_{1}(X)$ and ${g}_{2}(X)$. As a result of each iteration, the best values are determined, and the population is gathered around these values, and it is tried to obtain better values. Constraints are checked in both local and global search sections. In local search, energy values are calculated if these factors are suitable. If the new energy value is greater than the best value, the new value is assigned to the best value. If the best value found as a result of searches is greater than the current member, the new member is included in the population. The flowchart of the local search is given in Fig. 4.

The population size is one of the critical parameters in BA. It can be seen from previous studies^30^ that expanding the population reduces the number of iterations required and increases the probability of success. However, the number of function evaluations also rises. Having large numbers of elite sites does not significantly impact performance. The number of elite sites may be low, provided that it is non-zero^30^. The size of the scout bees population (*n)*, is usually chosen to be between 30 and 100. This study conducted two scenarios for 30 and 50 to determine the appropriate number (Table 2). Other parameters were determined depending on the population number. The number of selected sites (m) was (approximately) 25% of the population size, and the number of elite sites (e) among those selected was 25% of m. The number of foraging bees (search numbers) was selected as 100 for the elite sites and 30 for the other local sites. Neighborhood search is an essential concept for all evolutionary algorithms. In this study, the shrinking neighborhood method was used. This method decreases neighborhood size at a specific rate during each iteration. In future iterations, more precise searches can be done with smaller neighborhood values^30^.

**Table 2:** Parameters of BA.

Parameters	Scenario-1	Scenario-2
Number of iterations	30	50
Number of scout bees (n)	30	50
Number of bees for elite sites (nep)	30	30
Number of bees for selected sites (nsp)	10	10
Number of elite sites (e)	2	3
Number of selected sites (m)	8	12

### Optimization results

**Figure 5:**
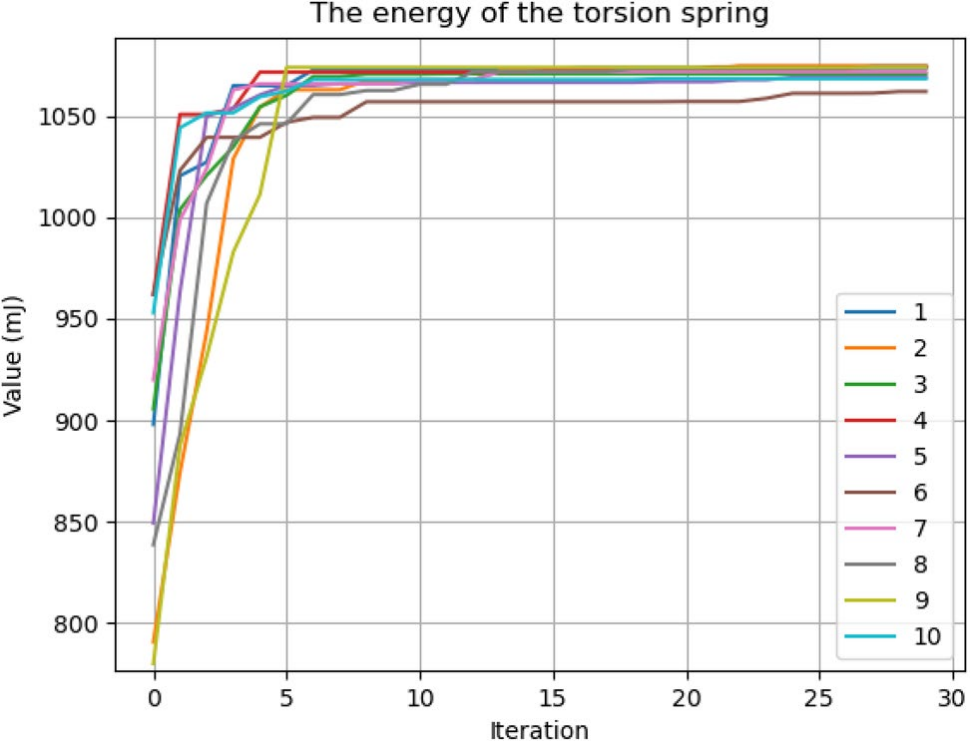
Optimization results for torsion spring—Scenario 1.

**Figure 6:**
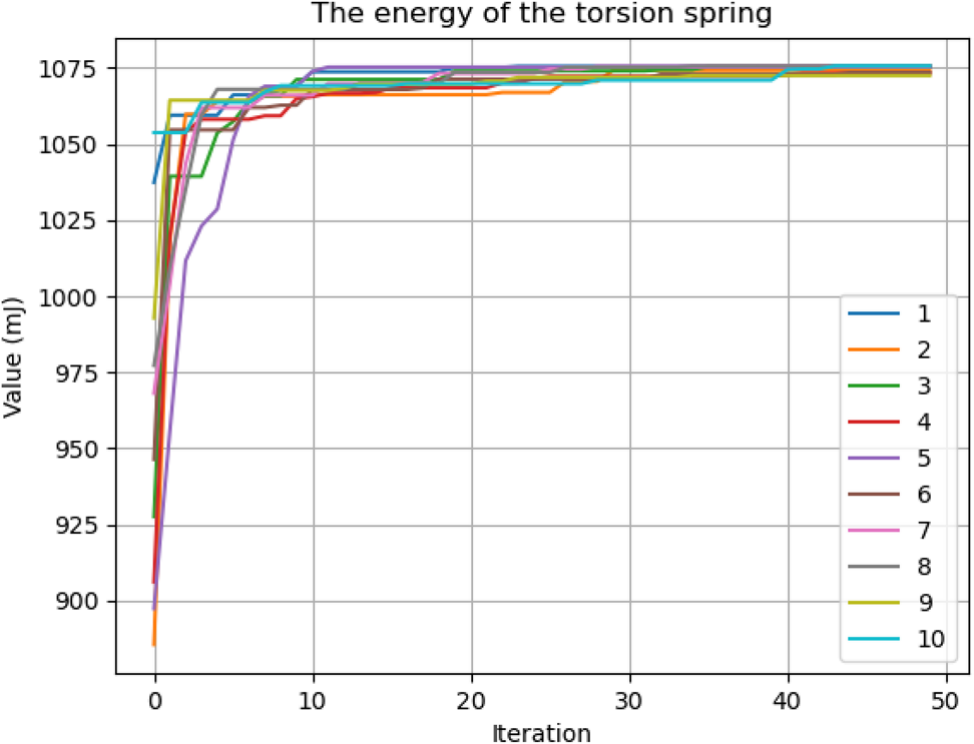
Optimization results for torsion spring—Scenario 2.

Ten consecutive tests were conducted with each scenario to test the repeatability of the optimization algorithm. Figure 5 shows the optimization results of the torsion spring for Scenario 1, and Fig. 6 shows Scenario 2. Test data are also given in Tables 3 and 4 (the table containing the results obtained for the compression spring is in the Supplementary Information S1). As seen from the graphs, there is a rapid increase in the first iterations before reaching the maximum value. The bee population intensifies its search around good values with the first iterations. In Scenario 1 results, some tests were below the maximum value. In Scenario 2, it is seen that all optimization results approach the maximum values by increasing the population and other related parameters. It is seen that the values in scenario 2 are sufficient for the algorithm.

**Table 3:** Optimization results for torsion spring—Scenario 1.

Parameters	1	2	3	4	5	6	7	8	9	10
d (mm)	1.7	1.7	1.7	1.7	1.7	1.7	1.7	1.7	1.7	1.7
Dm (mm)	20.59	20.89	19.95	20.87	20.40	18.23	19.98	20.80	20.64	19.44
N	4.98	4.91	5.16	4.91	5.05	5.69	5.15	4.93	4.97	5.3
Theta (°)	170	170	170	170	170	170	170	170	170	170
SFC (°)	170	170	170	170	170	170	170	170	170	170
Energy (mJ)	1073	1075	1071	1074	1069	1062	1072	1075	1074	1069

**Table 4:** Optimization results for torsion spring—Scenario 2.

Parameters	1	2	3	4	5	6	7	8	9	10
d (mm)	1.7	1.7	1.7	1.7	1.7	1.7	1.7	1.7	1.7	1.7
Dm (mm)	21	20.81	21	20.72	21	20.42	21	21	20.42	21
N	4.88	4.93	4.88	4.96	4.88	5.03	4.88	4.88	5.03	4.88
theta (°)	170	170	170	170	170	170	170	170	170	170
SFC (°)	170	170	170	170	170	170	170	170	170	170
Energy (mJ)	1075	1074	1075	1073	1075	1073	1075	1075	1073	1075

While obtaining maximum energy values in iterations, safety factors, which are the constraints of the study, are also provided. Safety factors are shown in the table. The energy values obtained by BA are compared with those obtained by the DOE method^5^ in Table 5. (For ease of production, the number of turns (N) is 4.9 instead of 4.88 in the torsion spring, and the deflection (xd) is 8 mm instead of 7.99 mm in the compression spring.) It is seen that BA has brought better results within the safety factors. BA evaluates all the values with both local and global searches. Thus, it can try more alternatives more quickly.

**Table 5:** Parameters of compression and torsion springs design.

Parameters and constraints	The Bees algorithm		Design of experiment	
	Compression spring	Torsion spring	Compression spring	Torsion spring
Wire diameter (d)	0.5 mm	1.7 mm	0.4 mm	1.6 mm
Coiling diameter (Dm)	3.6 mm	21 mm	3.6 mm	18 mm
Coiling number (N)	12	4.9	8.5	5.5
Deflection (xd/θ)	8 mm	170°	8.6 mm	160°
Safety factor (SFC)	1.2	–	1.24	–
Safety angle (SFT)	–	170°	–	160°
Energy (mJ)	37.24 mJ	1075 mJ	25 mJ	773.7 mJ

## Dynamic analysis of the wing mechanism and experimental results

In this study, Adams was used to analyzing the wing mechanism’s movements. First, the 3D model of the mechanism was transferred to Adams. Then, the springs whose parameters were selected in the previous section were defined. Also, some other parameters must be defined to make a realistic analysis. These are physical parameters such as joints, material specifications, contacts, frictions, and gravity forces. There is a revolution joint between the blade shaft and the bearings. There are 5–6 cylindrical joints. There are 5–1 fixed joint. The main body is made of aluminium material and is fixed. The material of other components is steel. The friction coefficients of the friction surfaces, contact stiffness, and penetration depth was selected according to the material type. (AISI 304 stainless steel) In this study, the critical parameter is the wing mechanism’s opening time, which should be less than 200 ms. Therefore, the wing opening time was observed in the analysis.

**Figure 7:**
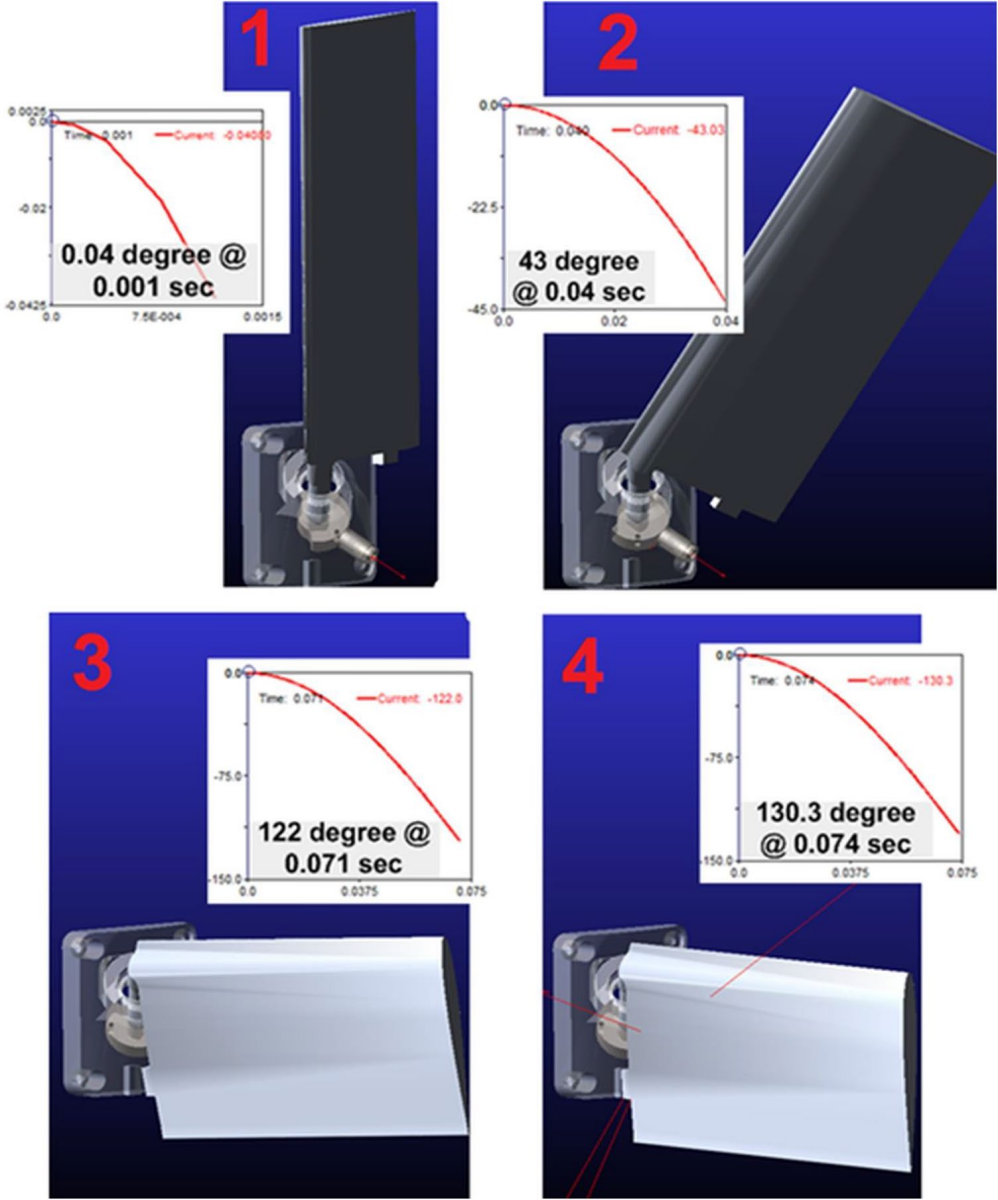
Dynamic simulation of the mechanism from folded to the deployed position.

As a result of the Adams analysis, the wing mechanism’s opening time was 74 ms. The results of dynamic simulation from 1 to 4 are shown in Fig. 7. The first picture of Fig. 5 shows the start time of the simulation, and the wing is waiting folded position. (2) shows the position of the wing after 40 ms, and at that moment, the wing has turned 43 degrees. (3) shows the position of the wing after 71 ms. Moreover, the last picture (4) shows the end of the wing’s turning and the opening position. As a result of the dynamic analysis, it was observed that the wing mechanism opened in a much shorter time than the target value of 200 ms. Moreover, safety constraints were selected from the highest values recommended in the literature when determining spring parameters.

After all the design, optimization, and simulation studies were completed, prototypes of the mechanism were manufactured and integrated. Then, tests of the prototypes were carried out in order to verify the simulation results. First, the main casing was fixed and the wing was folded. Then, the wing was released from the folded position, and the video record was taken while the wing was rotating from the folded position to the deployed position. A chronometer was also used during the video record to analyse the timing.

**Figure 8:**
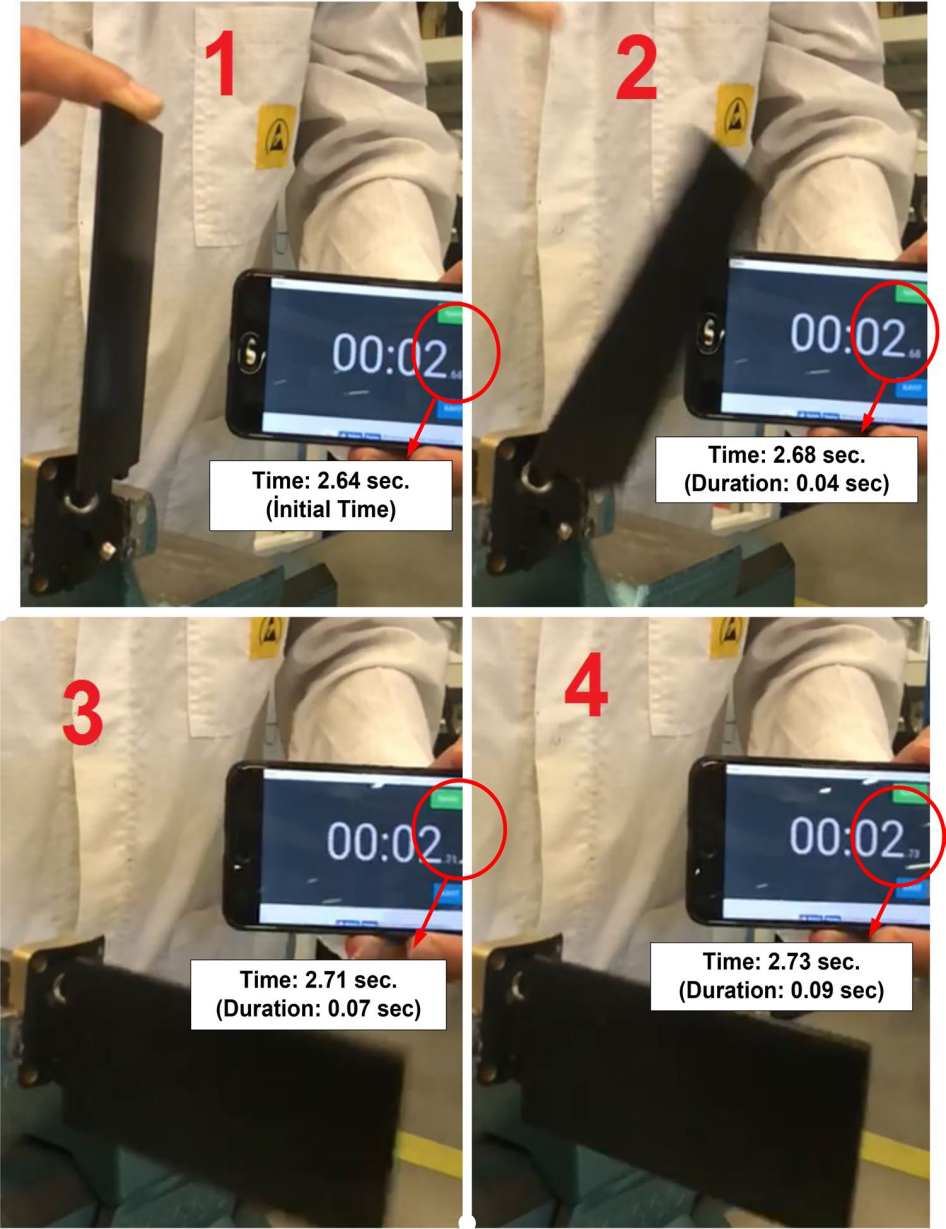
Prototype test for deployment time analysis of foldable wing mechanism.

Figure 8 presents frames from the video record numbered 1–4. The frame numbered 1 in the figure shows the moment the folded wing was released. That instant is taken to be the initial time t_0_. Frames numbered 2, and 3 show the wing position 40 ms and 70 ms after the initial time. When frames 3 and 4 were analyzed, it could be seen that stabilisation of the wing movement was achieved 90 ms after t_0_, and deployment of the wing was completed between 70 and 90 ms. This situation means that both the simulation and the prototype test give approximately the same deployment time of the missile wing, and the design meets the performance requirement of the mechanism.

## Conclusion

In this paper, the torsion and compression springs used in the foldable wing mechanism are optimized using BA. Parameters have been reached quickly with a low number of iterations. It is calculated as 1075 mJ for the torsion spring and 37.24 mJ for the compression spring. These values are 40–50% better than previous DOE studies. The springs were integrated into the mechanism and analyzed in the ADAMS program. In the analysis, it was seen that the wing opened within 74 ms. This value is well below the project target of 200 ms. In subsequent experimental studies, the opening time was measured as approximately 90 ms. This 16 ms difference between the analyzes may have been caused by environmental factors not modeled in the program. It is thought that the optimization algorithm obtained as a result of the study can also be used for different spring designs.

The material of the springs was predetermined, and it was not used as a variable in the optimization. Since there are many different types of springs in aircraft and missiles, BA will be applied to the designing other types of spring using different materials to achieve optimum spring designs in future studies.

### Ethics approval

We declare that this manuscript is original, has not been published before, and is not currently being considered for publication elsewhere.

## Supplementary Information

Supplementary Information 1.

Supplementary Information 2.

Supplementary Information 3.

## Data Availability

All data generated or analysed during this study are included in this published article [and its supplementary information files].

## Code availability

Algorithm codes have been uploaded into the system (Supplementary material).
