# Supplementary material for: Optimization of spring parameters by using the Bees algorithm for the foldable wing mechanism
Source: Sci Rep. 2022 Dec 19;12:21913. doi: 10.1038/s41598-022-26361-1 (PMC9763473; doi:10.1038/s41598-022-26361-1)
Supplement: Supplementary file 1 — Supplementary Information 1. [file 41598_2022_26361_MOESM1_ESM.docx]

**Optimization Results for Compression Spring**

**Supplementary Table** **S1** Optimization results for compression spring – Scenario 1

| **Parameters** | **1** | **2** | **3** | **4** | **5** | **6** | **7** | **8** | **9** | **10** |
| --- | --- | --- | --- | --- | --- | --- | --- | --- | --- | --- |
| d (mm) | 0.5 | 0.5 | 0.5 | 0.5 | 0.5 | 0.5 | 0.5 | 0.5 | 0.5 | 0.5 |
| Dm (mm) | 3.6 | 3.6 | 3.6 | 3.6 | 3.6 | 3.6 | 3.6 | 3.6 | 3.6 | 3.6 |
| N | 12 | 12 | 12 | 12 | 12 | 12 | 12 | 12 | 12 | 12 |
| xd (mm) | 7.98 | 7.99 | 7.97 | 7.99 | 7.99 | 7.99 | 7.98 | 7.99 | 7.98 | 7.98 |
| SFC | 1.2 | 1.2 | 1.2 | 1.2 | 1.2 | 1.2 | 1.2 | 1.2 | 1.2 | 1.2 |
| Energy (mJ) | 37.22 | 37.23 | 37.11 | 37.23 | 37.23 | 37.23 | 37.22 | 37.23 | 37.20 | 37.22 |


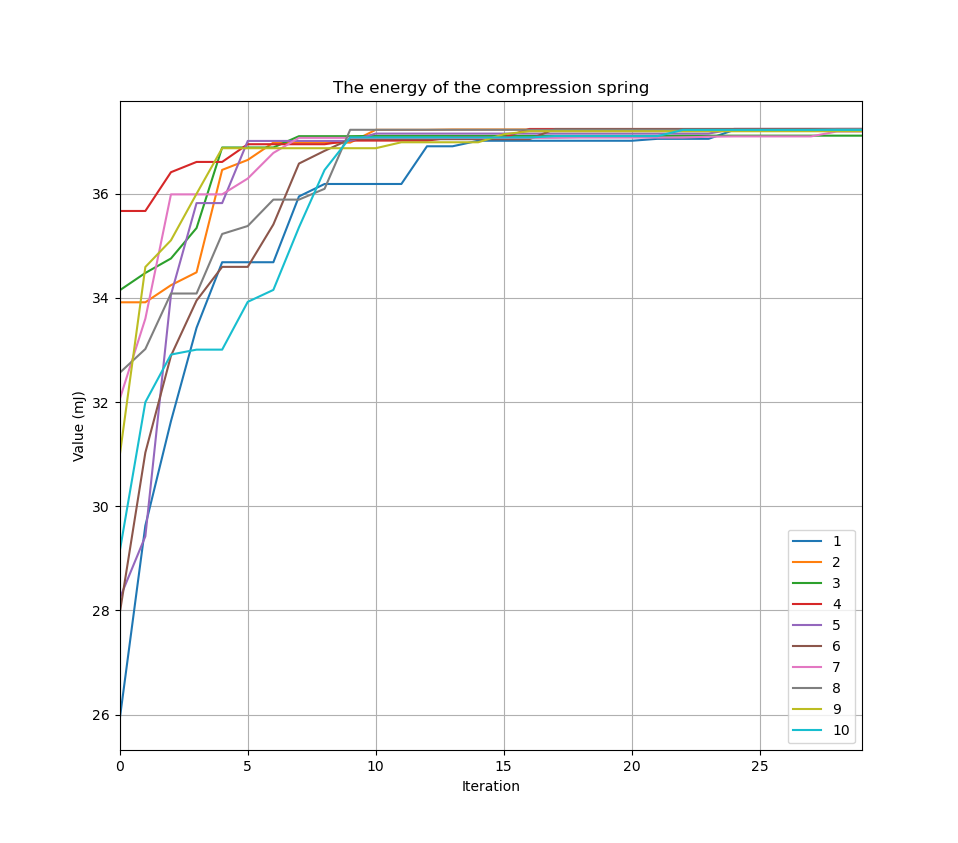


**Supplementary Figure S1.** Optimization results for compression spring – Scenario 1

**Supplementary Table** **S2** Optimization results for compression spring – Scenario 2

| **Parameters** | **1** | **2** | **3** | **4** | **5** | **6** | **7** | **8** | **9** | **10** |
| --- | --- | --- | --- | --- | --- | --- | --- | --- | --- | --- |
| d (mm) | 0.5 | 0.5 | 0.5 | 0.5 | 0.5 | 0.5 | 0.5 | 0.5 | 0.5 | 0.5 |
| Dm (mm) | 3.6 | 3.6 | 3.6 | 3.6 | 3.6 | 3.6 | 3.6 | 3.6 | 3.6 | 3.6 |
| N | 12 | 12 | 12 | 12 | 12 | 12 | 12 | 12 | 12 | 12 |
| xd (mm) | 7.99 | 7.99 | 7.99 | 7.99 | 7.99 | 7.99 | 7.99 | 7.99 | 7.99 | 7.99 |
| SFC | 1.2 | 1.2 | 1.2 | 1.2 | 1.2 | 1.2 | 1.2 | 1.2 | 1.2 | 1.2 |
| Energy (mJ) | 37.23 | 37.24 | 37.24 | 37.24 | 37.23 | 37.24 | 37.23 | 37.23 | 37.24 | 37.24 |


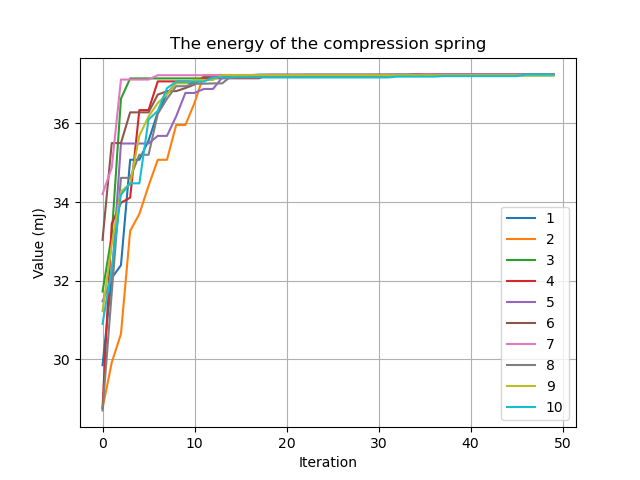


**Supplementary Figure S2.** Optimization results for compression spring – Scenario 2
