# Supplementary material for: Optimization of spring parameters by using the Bees algorithm for the foldable wing mechanism
Source: Sci Rep. 2022 Dec 19;12:21913. doi: 10.1038/s41598-022-26361-1 (PMC9763473; doi:10.1038/s41598-022-26361-1)
Supplement: Supplementary file 3 — Supplementary Information 3. [file 41598_2022_26361_MOESM3_ESM.docx]

**Python code for compression spring**

import matplotlib.pyplot as plt

import numpy as np

import random as rnd

"""Springs Energy Function"""

def springs_energy(d, xd, Dm, N):

G = 83.7E9

cost = (((d*0.001)**4) * G * ((xd*0.001)**2)) / (16*((Dm*0.001)**3) * N)

C = Dm / d

Kb = ((4*C) + 2)/((4*C) - 3)

Fs = ((xd*0.001) * ((d*0.001)**4) * G) / (8 * ((Dm*0.001)**3) * N)

Ts = (Kb * 8 * Fs * Dm*0.001) / (np.pi * ((d*0.001)**3))

safety = (980*1000000) / Ts

return cost*1000, safety

"""Neighborhood Function of d"""

def ngh_func_d(x, r_d):

new_d = x + rnd.uniform(-r_d, r_d)

if new_d <= 0.3:

new_d = 0.3

elif new_d >= 0.5:

new_d = 0.5

return new_d

"""Neighborhood Function of Dm"""

def ngh_func_Dm(x, r_Dm):

new_Dm = x + rnd.uniform(-r_Dm, r_Dm)

if new_Dm <= 3.1:

new_Dm = 3.1

elif new_Dm >= 3.6:

new_Dm = 3.6

return new_Dm

"""Neighborhood Function of N"""

def ngh_func_N(x, r_N):

new_N = x + rnd.uniform(-r_N, r_N)

if new_N <= 8:

new_N = 8

elif new_N >= 12:

new_N = 12

return new_N

"""Neighborhood Function of xd"""

def ngh_func_xd(x, r_xd):

new_xd = x + rnd.uniform(-r_xd, r_xd)

if new_xd <= 6:

new_xd = 6

elif new_xd >= 9:

new_xd = 9

return new_xd

"""Algorithm Parameters"""

itr_num = 50 # Maximum Number of Iterations

pop_num = 50 # Number of Scout Bees

el_sit = 3 # Number of Elite Sites

sel_sit = 12 # Number of Selected Sites

el_bee = 30 # Number of Recruited Bees for Elite Sites

sel_bee = 10 # Number of Recruited Bees for Selected Sites

r_d = 0.02 # d Neighborhood Radius

r_xd = 0.3 # xd Neighborhood Radius

r_Dm = 0.05 # Dm Neighborhood Radius

r_N = 0.4 # N Neighborhood Radius

rdamp = 1 # Neighborhood Radius Damp Rate

"""Problem Parameters"""

dmin = 0.3 # Wire diameter min.(mm)

dmax = 0.5 # Wire diameter max.(mm)

Dmmin = 3.1 # Coiling diameter min. (mm)

Dmmax = 3.6 # Coiling diameter max. (mm)

Nmin = 8 # Coiling Number min.

Nmax = 12 # Coiling Number max.

xdmin = 6 # Deflection min. (mm)

xdmax = 9 # Deflection max. (mm)

"""First Population"""

matr = []

k = 0

while k < pop_num:

d = rnd.uniform(dmin, dmax)

xd = rnd.uniform(xdmin, xdmax)

Dm = rnd.uniform(Dmmin, Dmmax)

N = rnd.uniform(Nmin, Nmax)

[cost, safety] = springs_energy(d, xd, Dm, N)

if safety >= 1.2:

matr.append([d, xd, Dm, N, cost, safety])

k += 1

pop_matr = np.reshape(matr,(pop_num, 6)) #Array to matrix

# Sorting population

population = pop_matr[np.argsort(-pop_matr[:, 4])]

print("\nBest solution in every iteration.")

bestbeelist = []

bestbee = []

"""Algorithm"""

for it in range(0, itr_num):

"""Elite Site Search"""

for enmb in range(0, el_sit):

dumy_tot = 0

bestnew_bee = [0, 0, 0, 0, 0, 0]

d = population[enmb][0]

xd = population[enmb][1]

Dm = population[enmb][2]

N = population[enmb][3]

for ebnmb in range(0, el_bee):

new_d = ngh_func_d(d, r_d)

new_xd = ngh_func_xd(xd, r_xd)

new_Dm = ngh_func_Dm(Dm, r_Dm)

new_N = ngh_func_N(N, r_N)

[new_cost, new_safety] = springs_energy(new_d, new_xd, new_Dm, new_N)

new_bee = [new_d, new_xd, new_Dm, new_N, new_cost, new_safety]

if new_bee[4] > dumy_tot and new_bee[5] >= 1.2:

dumy_tot = new_bee[4]

bestnew_bee = new_bee

if bestnew_bee[4] > population[enmb][4]:

population[enmb] = bestnew_bee

"""Selected Site Search"""

for snmb in range(el_sit, sel_sit):

dumy_tot = 0

bestnew_bee = [0, 0, 0, 0, 0, 0]

d = population[snmb][0]

xd = population[snmb][1]

Dm = population[snmb][2]

N = population[snmb][3]

for ebnmb in range(0, sel_bee):

new_d = ngh_func_d(d, r_d)

new_xd = ngh_func_xd(xd, r_xd)

new_Dm = ngh_func_Dm(Dm, r_Dm)

new_N = ngh_func_N(N, r_N)

[new_cost, new_safety] = springs_energy(new_d, new_xd, new_Dm, new_N)

new_bee = [new_d, new_xd, new_Dm, new_N, new_cost, new_safety]

if new_bee[4] > dumy_tot and new_bee[5] >= 1.2:

dumy_tot = new_bee[4]

bestnew_bee = new_bee

if bestnew_bee[4] > population[snmb][4]:

population[snmb] = bestnew_bee

"""Global Search"""

k = 0

while k < (pop_num - sel_sit - 1):

d = rnd.uniform(dmin, dmax)

xd = rnd.uniform(xdmin, xdmax)

Dm = rnd.uniform(Dmmin, Dmmax)

N = rnd.uniform(Nmin, Nmax)

[cost, safety] = springs_energy(d, xd, Dm, N)

if safety >= 1.2 and safety <= 1.5:

k += 1

population[sel_sit + k] = [d, xd, Dm, N, cost, safety]

"""Sort population"""

population = population[np.argsort(-population[:, 4])]

"""ngh Update"""

r_d = r_d * rdamp

r_xd = r_xd * rdamp

r_Dm = r_Dm * rdamp

r_N = r_N * rdamp

"""Best solution"""

print(population[0])

bestbeelist.append(population[0][4])

bestbee.append(population[0])

print("\nBest solution.")

result = bestbee[itr_num-1]

print(result)

plt.plot(bestbeelist)

plt.title("The energy of the compression spring")

plt.xlabel('Iteration')

plt.ylabel('Value (mJ)')

plt.grid()

plt.show()

**Python code for torsion spring**

import matplotlib.pyplot as plt

import numpy as np

import random as rnd

"""Springs Energy Function"""

def springs_energy(d, th, Dm, N):

E = 203.4E9

Sy = 1600E6

cost = (((d*0.001)**4) * E * ((th)**2) * (np.pi/180)) / (7776 * ((Dm*0.001)) * N)

C = Dm / d

Ki = ((4 * (C**2)) - C - 1) / (4*C * (C - 1))

M = (np.pi * ((d*0.001)**3) * Sy) / (32 * Ki)

k1 = (((d*0.001)**4) * E) / (10.8 * ((Dm*0.001)) * N)

safety = ((M * 360) / k1)

return cost*1000, safety

"""Neighborhood Function of d"""

def ngh_func_d(x, r_d):

new_d = x + rnd.uniform(-r_d, r_d)

if new_d <= 1:

new_d = 1

elif new_d >= 1.7:

new_d = 1.7

return new_d

"""Neighborhood Function of Dm"""

def ngh_func_Dm(x, r_Dm):

new_Dm = x + rnd.uniform(-r_Dm, r_Dm)

if new_Dm <= 15:

new_Dm = 15

elif new_Dm >= 21:

new_Dm = 21

return new_Dm

"""Neighborhood Function of N"""

def ngh_func_N(x, r_N):

new_N = x + rnd.uniform(-r_N, r_N)

if new_N <= 3:

new_N = 3

elif new_N >= 7:

new_N = 7

return new_N

"""Neighborhood Function of theta"""

def ngh_func_th(x, r_th):

new_th = x + rnd.uniform(-r_th, r_th)

if new_th <= 130:

new_th = 130

elif new_th >= 170:

new_th = 170

return new_th

"""Algorithm Parameters"""

itr_num = 50 # Maximum Number of Iterations

pop_num = 50 # Number of Scout Bees

el_sit = 3 # Number of Elite Sites

sel_sit = 12 # Number of Selected Sites

el_bee = 30 # Number of Recruited Bees for Elite Sites

sel_bee = 10 # Number of Recruited Bees for Selected Sites

r_d = 0.07 # d Neighborhood Radius

r_th = 4 # theta Neighborhood Radius

r_Dm = 0.6 # Dm Neighborhood Radius

r_N = 0.4 # N Neighborhood Radius

rdamp = 1 # Neighborhood Radius Damp Rate

"""Problem Parameters"""

dmin = 1 # Wire diameter min.(mm)

dmax = 1.7 # Wire diameter max.(mm)

Dmmin = 15 # Coiling diameter min. (mm)

Dmmax = 21 # Coiling diameter max. (mm)

Nmin = 3 # Coiling Number min.

Nmax = 7 # Coiling Number max.

thmin = 130 # Deflection min. (mm)

thmax = 170 # Deflection max. (mm)

"""First Population"""

matr = []

k = 0

while k < pop_num:

d = rnd.uniform(dmin, dmax)

th = rnd.uniform(thmin, thmax)

Dm = rnd.uniform(Dmmin, Dmmax)

N = rnd.uniform(Nmin, Nmax)

[cost, safety] = springs_energy(d, th, Dm, N)

if safety >= th:

matr.append([d, th, Dm, N, cost, safety])

k += 1

pop_matr = np.reshape(matr,(pop_num, 6)) #Array to matrix

# Sorting population

population = pop_matr[np.argsort(-pop_matr[:, 4])]

print("\nBest solution in every iteration.")

bestbeelist = []

bestbee = []

"""Algorithm"""

for it in range(0, itr_num):

"""Elite Site Search"""

for enmb in range(0, el_sit):

dumy_tot = 0

bestnew_bee = [0, 0, 0, 0, 0, 0]

d = population[enmb][0]

th = population[enmb][1]

Dm = population[enmb][2]

N = population[enmb][3]

for ebnmb in range(0, el_bee):

new_d = ngh_func_d(d, r_d)

new_th = ngh_func_th(th, r_th)

new_Dm = ngh_func_Dm(Dm, r_Dm)

new_N = ngh_func_N(N, r_N)

[new_cost, new_safety] = springs_energy(new_d, new_th, new_Dm, new_N)

new_bee = [new_d, new_th, new_Dm, new_N, new_cost, new_safety]

if new_bee[4] > dumy_tot and new_bee[5] >= new_th:

dumy_tot = new_bee[4]

bestnew_bee = new_bee

if bestnew_bee[4] > population[enmb][4]:

population[enmb] = bestnew_bee

"""Selected Site Search"""

for snmb in range(el_sit, sel_sit):

dumy_tot = 0

bestnew_bee = [0, 0, 0, 0, 0, 0]

d = population[snmb][0]

th = population[snmb][1]

Dm = population[snmb][2]

N = population[snmb][3]

for ebnmb in range(0, sel_bee):

new_d = ngh_func_d(d, r_d)

new_th = ngh_func_th(th, r_th)

new_Dm = ngh_func_Dm(Dm, r_Dm)

new_N = ngh_func_N(N, r_N)

[new_cost, new_safety] = springs_energy(new_d, new_th, new_Dm, new_N)

new_bee = [new_d, new_th, new_Dm, new_N, new_cost, new_safety]

if new_bee[4] > dumy_tot and new_bee[5] >= new_th:

dumy_tot = new_bee[4]

bestnew_bee = new_bee

if bestnew_bee[4] > population[snmb][4]:

population[snmb] = bestnew_bee

"""Global Search"""

k = 0

while k < (pop_num - sel_sit - 1):

d = rnd.uniform(dmin, dmax)

th = rnd.uniform(thmin, thmax)

Dm = rnd.uniform(Dmmin, Dmmax)

N = rnd.uniform(Nmin, Nmax)

[cost, safety] = springs_energy(d, th, Dm, N)

if safety >= th:

k += 1

population[sel_sit + k] = [d, th, Dm, N, cost, safety]

"""Sort population"""

population = population[np.argsort(-population[:, 4])]

"""ngh Update"""

r_d = r_d * rdamp

r_th = r_th * rdamp

r_Dm = r_Dm * rdamp

r_N = r_N * rdamp

"""Best solution"""

print(population[0])

bestbeelist.append(population[0][4])

bestbee.append(population[0])

print("\nBest solution.")

result = bestbee[itr_num-1]

print(result)

plt.plot(bestbeelist)

plt.title("The energy of the torsion spring")

plt.xlabel('Iteration')

plt.ylabel('Value (mJ)')

plt.grid()
